# A tin(iv) oxides/carbon nanotubes composite with core-tubule structure as an anode material for high electrochemistry performance LIBs

**DOI:** 10.1039/c8ra00346g

**Published:** 2018-04-10

**Authors:** Yu Ji, Li Li, Yang Zhenyu, Cai Jianxin

**Affiliations:** College of Chemistry, Nanchang University No. 999 Xuefu Road, New District of Honggutan Nanchang 330031 PR China yuji@ncu.edu.cn +86-791-83969514

## Abstract

SnO_2_/CNTs composites with core-tubule structure are prepared by a facile wet chemical method. The investigation of electrochemical characteristics of the SnO_2_/CNTs composites shows that the composites exhibit some advantages, such as stable core-tubule structure, small particle size of SnO_2_, low electron-transfer resistance and faster lithium ion migration speed. The final product synthesized under optimized conditions can release a stable capacity of about 743 mA h g^−1^ after 100 cycles at the current density of 0.4 A g^−1^, 598 mA h g^−1^ after 500 cycles at the current density of 4 A g^−1^. Even at a super high current density of 8 A g^−1^, the composite can still deliver a steady capacity of 457 mA h g^−1^, and the discharge capacity can be restored to 998 mA h g^−1^ when current density is decreased to 0.4 A g^−1^.

## Abstract 2

SnO_2_/CNTs composites with core-tubule structure are prepared by a facile wet chemical method.

## Graphical abstract

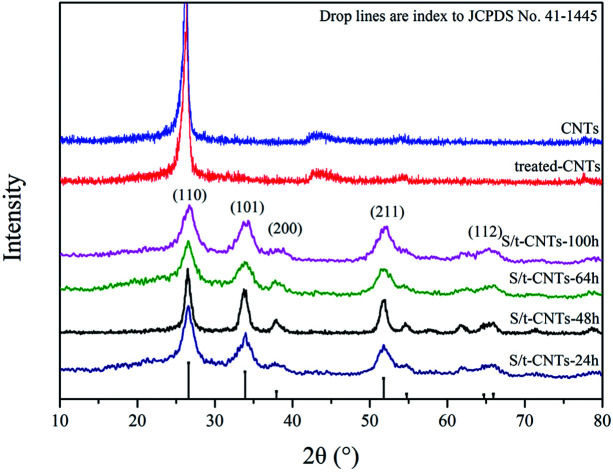

## 1. Introduction

Carbon nanotubes (CNTs) have much promise for application in LIBs owing to the high conductivity, high specific surface area, good permeability properties of lithium and stable electrochemistry.^1–3^ These properties made CNTs an optimum support matrix for composites.^4,5^ However, CNTs displayed a low charge & discharge capacity as an anode material for LIBs which limited its application in lithium-stored materials.^6^ Many scholars believed that a crafted combination of CNTs and high-capacity lithium-stored compounds like Sn, Cu, Si, Fe, Ni or their oxides and so on may work to display both high capacity and good cyclability.^7–11^

SnO_2_-based materials are perceived as attractive anode materials for LIBs due to their high theoretical capacity, environmental benignity, and widespread availability.^12^ However, the high discharge capacity is usually accompanied with large volumetric change during charging and discharging, which results in rapid capacity decay of the active materials.^13^ Two major methods have been developed to enhance the structural stability and electrochemical performance of SnO_2_-based anodes. The first is based on structuring the SnO_2_-based materials themselves, such as SnO_2_ nanoparticles,^14^ nanowires,^15^ nanotubes,^16^ hollow nanospheres,^17^ nanocubes^18^ and nanosheets.^19^ The other complementary method is to employ flexible carbon coatings, which act as a soft matrix, to accommodate the mechanical stress caused by volume expansion.^20,21^ A series of SnO_2_/CNTs composites have been designed and developed in recent years, such as tin oxides adsorbed on the surface of CNTs^22^ and SnO_2_@carbon hollow spheres.^23,24^ It suggests that CNTs can not only relieve the volume change, inhibiting aggregation, but also enhance the conductivity of the composites.^25,26^ Moreover, many efforts have been devoted to fill foreign materials into the hollow cavity of CNTs.^27,28^ Usually electrochemical deposition, solution-chemistry method and capillary forces action (physical deposition) are regarded as the commonly used techniques.^29,30^

Herein, we reported a special structure of SnO_2_/CNTs core-tubule composite and studied the effect of reaction times on the electrochemical performance of the composite as anode materials for LIBs.

## 2. Experimental

CNTs were provided by Nanjing Ji Bin Nanometer Science and Technology Co., Ltd. (Nanjing, China) and used as received. The inner and outer diameters of the CNTs were 20–50 nm and 30–60 nm measured by TEM (JEM-200CX, operated at 200 kV). CNTs were treated before used as the container of SnO_2_. A typical procedure was described as follows: the CNTs were dispersed in distilled water by ultrasonic wave for half an hour and then soaked in 30% hydrogen peroxide for 5 h.^31^ After that the CNTs were refluxed in nitric acid solution (20 wt%) at 100 °C for another 5 h. In the end, the treated-CNTs were rinsed and dried at 80 °C and then the open-tips CNTs were obtained (see the Fig. 2(a) and (b)).

**Fig. 1:**
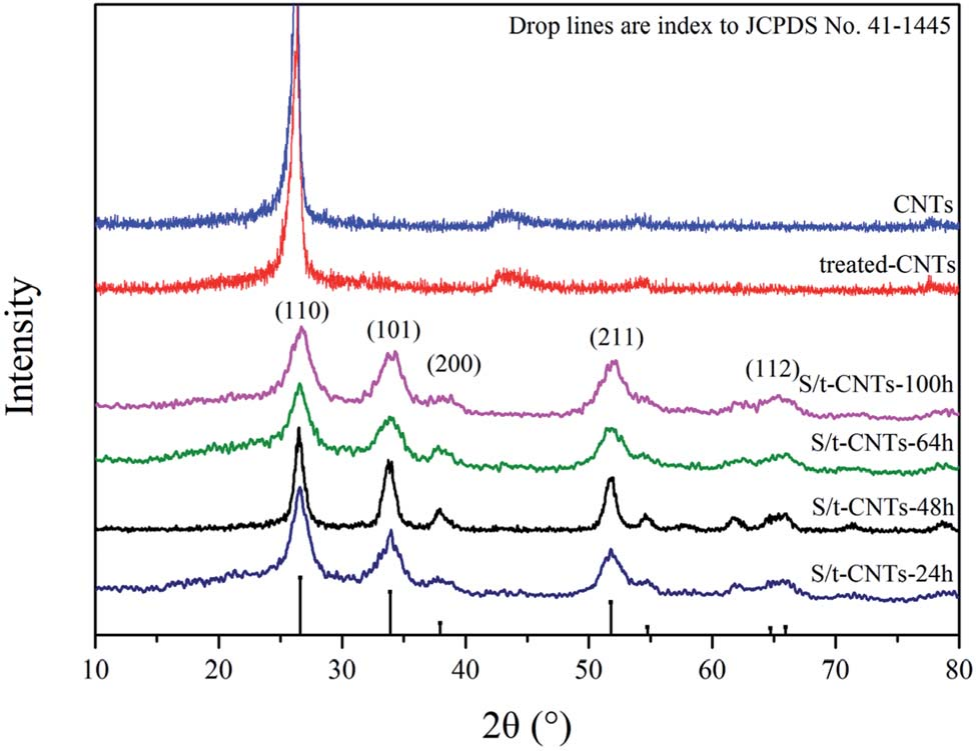
XRD patterns of S/t-CNTs-24 h, -48 h, -64 h, -100 h, treated-CNTs and CNTs.

**Fig. 2:**
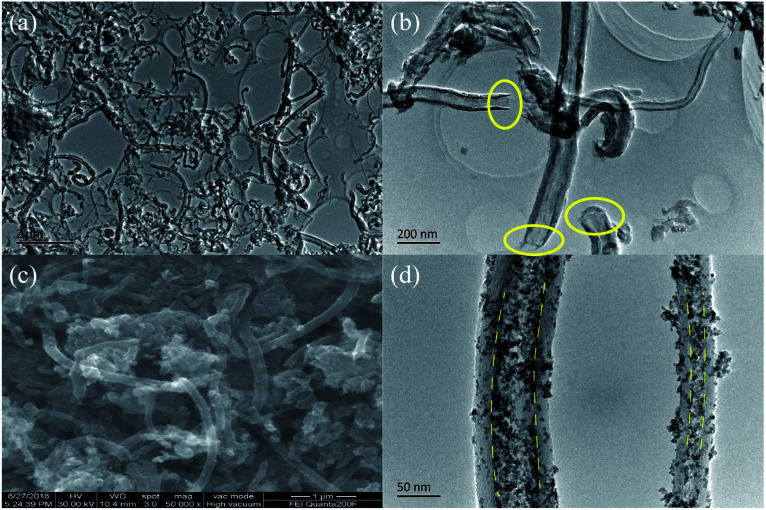
TEM images of treated-CNTs (a) and (b), SEM image (c) and TEM image (d) of the final product S/t-CNTs-100 h composite.

About 100 mg treated-CNTs were putted into a round bottom flask. Then the flask was evacuated and a aqueous solution of SnCl_2_ and 0.5 ml HCl (37%) was added into the flask by an injector. The mixture was stirred at room temperature and the flask was maintained vacuum state for 8 h, 16 h, 24 h, 48 h, 64 h, 72 h and 100 h to complete the filling process. After that the solution were refluxed at 140 °C for 3 h, and the black samples were collected and washed by ethanol for several times to remove the attachments of outside of CNTs as far as possible. The samples were dried at 60 °C for 12 h, and then calcined at 400 °C for 2 h under the protection of argon. The final samples were marked as S/t-CNTs-8 h, S/t-CNTs-16 h and so on according the reaction time.

The as-samples were characterized by X-ray powder diffraction (XRD) using a Rigaku D/Max-IIIA X-ray diffractometer with Cu Kα radiation (*λ* = 1.54178 Å). Morphology and structure of the as-samples were examined by scanning electron microscopy (SEM, FEI Quanta 200F) and transmission electron microscopy (TEM, JEM-200CX). Thermogravimetric analysis (TGA) was carried out on a thermogravimetric-differential scanning calorimetry (TG-DSC) analyzer (Mettler-Toledo, TGA/DSCI-LF-1100) in air from room temperature to 1000 °C with a heating rate of 5 °C min^−1^.

Electrochemical measurements were carried out using coin-type half cells (CR2025 type) with lithium metal as the counter electrode. The working electrodes consisted of 84 wt% active material (*e.g.*, S/t-CNTs-8 h, about 0.5 g), 10 wt% conductive carbon (superP-Li, Timcal), 3 wt% carboxymethyl cellulose sodium (CMC) and 3 wt% styrene butadiene rubber (SBR). The mixed slurry was pasted on a copper foil and cut into small discs. The electrolyte was 1.3 M LiPF_6_ in ethylene carbonate (EC) and diethyl carbonate (DEC) with the volume ratio of EC/DEC = 3 : 1. The cells were assembled in a glove box filled with pure argon in the presence of an oxygen scavenger and a sodium-drying agent and then aged for 12 h at room temperature before the electrochemical test between 0.01 V and 2.5 V (*vs.* Li/Li^+^). Cyclic voltammetry (CV) and electrochemical impedance spectra (EIS) measurements were recorded by the electrochemical workstation system IM6ex (Zahner).

## 3. Results and discussion

Fig. 1 shows the X-ray diffraction (XRD) patterns of the selected samples S/t-CNTs-24 h, S/t-CNTs-48 h, S/t-CNTs-64 h, S/t-CNTs-100 h, CNTs and treated-CNTs. The (002) diffraction peak of graphite can be obviously observed from the XRD patterns of the CNTs and treated-CNTs. The diffraction peaks at 2*θ* = 26.7°, 34.1°, 51.5°, 65.5° can be assigned to 110, 101, 211 and 112 planes of the cassiterite SnO_2_, respectively. No impurities such as SnCl_2_ and SnO were found. The highly broad diffraction peaks indicate the nanocrystalline nature of the composites, which will contribute to the high rate electrochemical performance of the composites. The mean particle size of the SnO_2_ particles estimated from Scherer's formula is about 4–5 nm agreeing with the TEM results in the follow discussion.

After the oxidation by the hydrogen peroxide and nitric acid solution, the 1D tubular structure of CNTs is retained. The long CNTs are cut down to short tubes and most tips are opened, seen in Fig. 2(a) and (b). Fig. 2(c) and (d) show the SEM and TEM images of the final product S/t-CNTs-100 h. Both SEM and TEM images confirm that the outer surfaces of CNTs are covered with few SnO_2_ particles. But most of SnO_2_ particles were filled in the hollow cavity of CNTs indicated by the clear boundary, seen in Fig. 2(d). Otherwise, from the image Fig. 2(d), it can be seen that the size distribution of the SnO_2_ particles is very uniform, and the average diameter is about 4∼5 nm which is consistent with the result calculated from the XRD data.

**Fig. 3:**
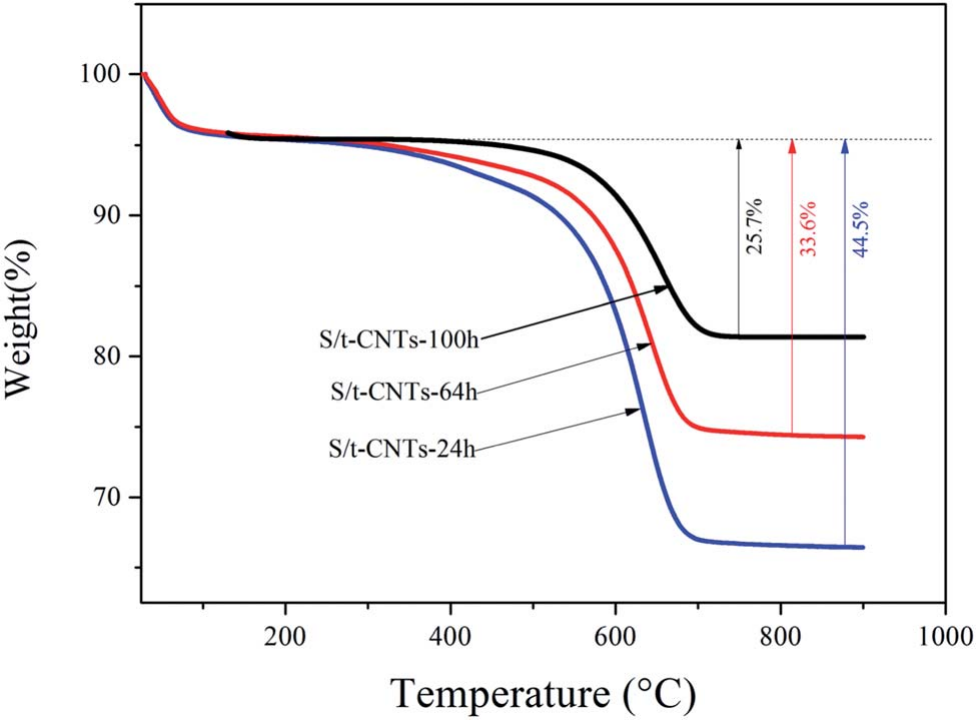
TG curves of the final products S/t-CNTs-24 h, -64 h and -100 h.

The actual content of SnO_2_ in SnO_2_/CNTs composites was analyzed by TG method, the result is showed in Fig. 3. All samples were heated from room temperature to 1000 °C in air atmosphere. From the Fig. 3, it can be seen that the main mass loss occurred in the range of 200–800 °C, which is caused by the combustion of CNTs in the composite.^28,30^ So, the content of SnO_2_ in the SnO_2_/CNTs composite is about 74.3 wt%, 66.4 wt% and 55.3 wt% respectively when the reaction time is 100 h, 64 h and 24 h. These data suggest that the content of SnO_2_ filled in the cavity of CNTs will increase with the increase of reaction time, which will result in the increase of charge & discharge capacity of the composite.

**Fig. 4:**
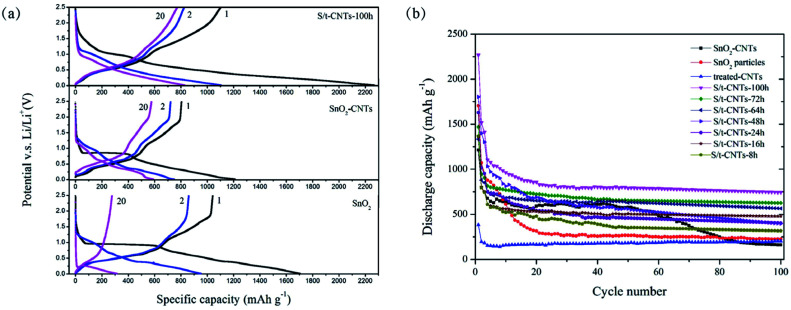
The selected charge & discharge curves (a) and cycle performance (b) of S/t-CNTs composites, SnO_2_-CNTs and SnO_2_ working at current density of 0.4 A g^−1^.

For comparison, the selected charge & discharge profiles of S/t-CNTs-100 h composite, SnO_2_-CNTs (SnO_2_ particles and CNTs were mixed simply by grinding) and bare SnO_2_ particles at a current density of 0.4 A g^−1^ between the potential range of 0.01–2.5 V (*vs.* Li/Li^+^) are shown in Fig. 4(a). The discharge capacity was calculated based on the mass of active materials (S/t-CNTs-100 h composite, SnO_2_-CNTs or SnO_2_). The initial discharge capacity of the S/t-CNTs-100 h composite is greater than 2200 mA h g^−1^, and it decrease to 1105 mA h g^−1^ in the next cycle. The reasons of the serious irreversible capacity loss are mainly attributed to the reduction of SnO_2_ by Li metal,^32^ the formation of SEI films and the side reactions between electrolyte and the functional groups on the surface of CNTs. The case of sample SnO_2_-CNTs and bare SnO_2_ are the same as for S/t-CNTs-100 h, but their initial discharge capacity are much lower than that of S/t-CNTs-100 h composite and decrease very quickly. After 20 cycles, the S/t-CNTs-100 h composite can still deliver a high discharge capacity of 829 mA h g^−1^, while sample SnO_2_-CNTs and SnO_2_ can only release a much lower discharge capacity of 585 mA h g^−1^ and 313 mA h g^−1^.

Fig. 4(b) shows the cycling performance of S/t-CNTs-100 h, -72 h, -64 h, -48 h, -24 h, -16 h, -8 h composites, SnO_2_-CNTs, SnO_2_ particles and treated-CNTs working at a current density of 0.4 A g^−1^ between 0.01–2.5 V. All the S/t-CNTs composites show an excellent cycling performance and the discharge capacity increase gradually with the increase of the reaction time means the content of SnO_2_ in the composite increased with the increase of the reaction time, which is agreement with the result of TG characterization. Sample S/t-CNTs-100 h can deliver high discharge capacity of 743 mA h g^−1^ after 100 cycles. The bare SnO_2_ particles can only deliver a discharge capacity of 225 mA h g^−1^ after 100 cycles, just a little higher than that of treated-CNTs. It is worth noting that sample SnO_2_-CNTs can release a high discharge capacity of 617 mA h g^−1^ in the first few cycles, which is higher than that of sample S/t-CNTs-48 h, -24 h, -16 h and -8 h. However, after 50 cycles its discharge capacity begin to decay sharply and only remain 162 mA h g^−1^ at the 100^th^ cycle. This should be caused by the huge and iterative volume change in the process of charge & discharge which will result in the failure of the electrode. Therefore, SnO_2_ particles mixing with CNTs by simply grinding cannot improve its electrochemical properties for long cycle.

The improved lithium storage performance of S/t-CNTs composites can be explained by the effect of small size of SnO_2_ and the restriction of CNTs.^33,34^ Ultra-small SnO_2_ particles can make the diffusion pathway of the Li-ions shorten^35,36^ and also make electrolyte diffuse better. On the other hand, the aggregation and large volume change of SnO_2_ during the process of charge & discharge is restricted by the unique tubular structure of CNTs.^37,38^

**Fig. 5:**
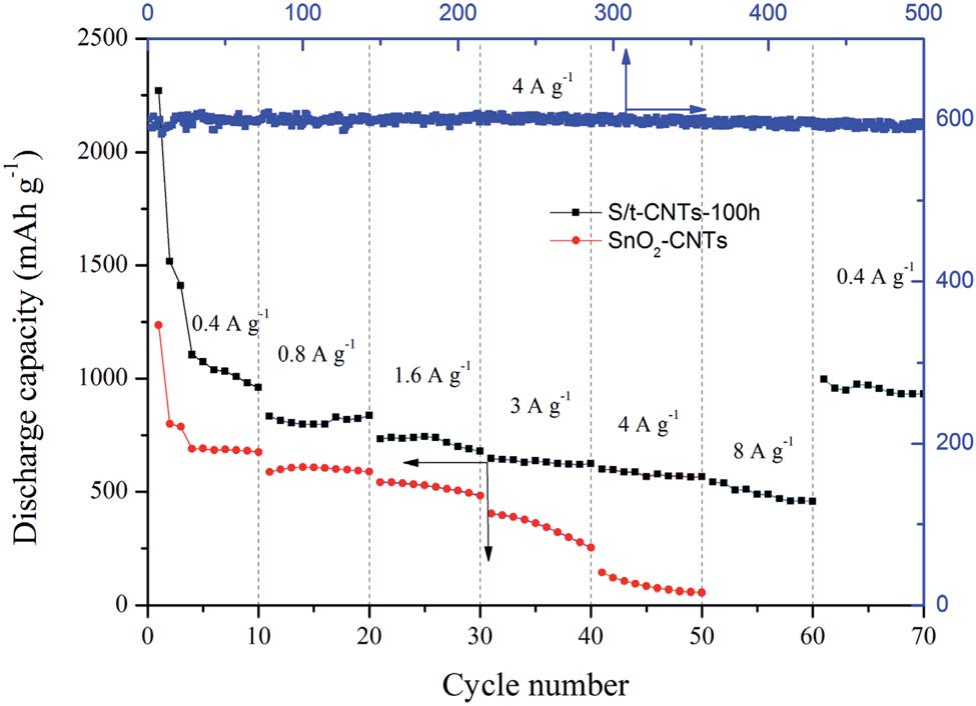
Rate capability of the S/t-CNTs-100 h composite and SnO_2_-CNTs; and cycle performance at current density of 4 A g^−1^ of sample S/t-CNTs-100 h.

The electrochemical performance of S/t-CNTs-100 h composite and SnO_2_-CNTs at different current density is given in Fig. 5. The reversible discharge capacities of S/t-CNTs-100 h are 962 mA h g^−1^, 837 mA h g^−1^, 680 mA h g^−1^, 625 mA h g^−1^ and 566 mA h g^−1^ after successively working at a current density of 0.4 A g^−1^, 0.8 A g^−1^, 1.6 A g^−1^, 3 A g^−1^ and 4 A g^−1^ for 10 cycles, respectively. While, the SnO_2_-CNTs delivers discharge capacity of 676 mA h g^−1^, 588 mA h g^−1^, 484 mA h g^−1^, 253 mA h g^−1^ and 56 mA h g^−1^ when cycled at 0.4 A g^−1^, 0.8 A g^−1^, 1.6 A g^−1^, 3 A g^−1^ and 4 A g^−1^ for 10 cycles, respectively. The rate performance of S/t-CNTs-100 h composite is much better than that of SnO_2_-CNTs. Even when the current density increased to 8 A g^−1^, the composite can still deliver a discharge capacity of 458 mA h g^−1^. And when the current density decreased to 0.4 A g^−1^, a greatly high discharge capacity of 998 mA h g^−1^ can be retained, revealing the nature of good conductivity of the composite. The curve of 500 cycles of S/t-CNTs-100 h composite working at a current density of 4 A g^−1^ is illustrated in the Fig. 5 too. It can be seen the discharge capacity of the composite is almost no attenuation, which means the excellent cycling stabilization of the composite in the long cycle. It is believed that the excellent performance at different current density and the long life are mainly benefit from the special core-tubule structure of the S/t-CNTs composite. CNTs can limit the huge and reiterative volume change of SnO_2_ and provide a good conductive network for the anode in the same time during the process of charge & discharge.^39,40^

**Fig. 6:**
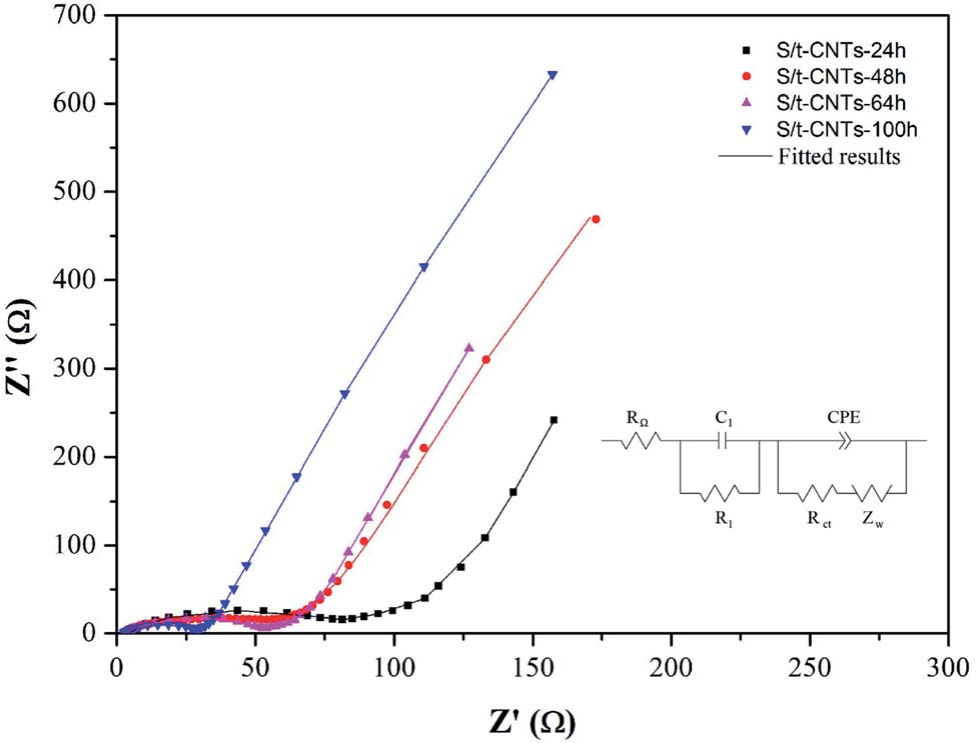
EIS curves of sample S/t-CNTs-24 h, -48 h, -64 h and -100 h composite.

Further investigation of the effect of reaction time on the electron transfer resistance of the S/t-CNTs composites by the EIS method is illustrated in Fig. 6. The equivalent circuit is shown in Fig. 6 too, where *R*_Ω_, *R*_l_, *R*_ct_, *Z*_w_ and CPE are corresponded to the electrolyte resistance, SEI resistance, charge-transfer resistance,^29,41^ Warburg impedance associated with the diffusion of Li^+^ and constant phase element related to double layer capacitance, respectively.^35,36,42^ The fitting results are listed in Table 1. As can be seen, with the increase of reaction time, *R*_l_ and *R*_ct_ are both decrease. That is, a longer reaction time will make more SnO_2_ nanoparticles enter into the cavity of CNTs. And the contact between the SnO_2_ nanoparticles or SnO_2_ nanoparticles and CNTs become more closely result in the decrease of resistance and the high electrochemical performance of the S/t-CNTs composites.

**Table 1:** Equivalent circuit parameters of the sample S/t-CNTs-24 h, -48 h, -64 h and -100 h

Samples	*R*_Ω_/Ω cm^2^	*R*_1_/Ω cm^2^	*R*_ct_/Ω cm^2^
S/t-CNTs-24 h	5.51	78.72	43.41
S/t-CNTs-48 h	2.54	54.20	55.45
S/t-CNTs-64 h	3.75	52.01	29.37
S/t-CNTs-100 h	2.88	29.61	24.05

## 4. Conclusion

A series of S/t-CNTs composites with different reaction times were prepared by a facile method. The results indicated that the amount of SnO_2_ nanoparticles filled in the hollow cavity of the CNTs will increase with the increase of reaction time. At the same time, the electrochemical performance of S/t-CNTs composites would become better because of the special core-tubule structure. The composite with reaction time 100 h released an ultra-high discharge capacity of 2200 mA h g^−1^ in the first cycle and maintained at about 829 mA h g^−1^ after about 20 cycles at a current density of 0.4 A g^−1^. Even when the current density increased to 8 A g^−1^, the composite can still deliver a steady capacity of 458 mA h g^−1^ and has a long cycle life.

## Conflicts of interest

There are no conflicts to declare.

## Supplementary Material
